# LONGITUDINAL CHANGE OF PHYSICAL AND COGNITIVE FUNCTIONS IN BLSA

**DOI:** 10.1093/geroni/igz038.2147

**Published:** 2019-11-08

**Authors:** Pei-Lun Kuo, Morgan Levine, Michelle Shardell, Susan Resnick, Eleanor M Simonsick, Jennifer A Schrack

**Affiliations:** 1 Johns Hopkins University Bloomberg School of Public Health, Baltimore, Maryland, United States; 2 Yale Center for Research on Aging (Y-Age), Department of Pathology, Yale School of Medicine, New Haven, Connecticut, United States; 3 Longitudinal Studies Section, Translational Gerontology Branch, National Institute On Aging , NIH, Baltimore, Maryland, United States; 4 Laboratory Of Behavioral Neuroscience, National Institute On Aging , NIH, Baltimore, Maryland, United States

## Abstract

Optimally integrating metrics of aging requires evaluating the metrics’ change with aging. We investigated longitudinal changes of physical and cognitive functions in the Baltimore Longitudinal Study of Aging. Usual gait speed declined -0.08 m/s (p<0.001) per decade at age 60 years, -0.10 m/s (p<0.001) per decade at 65 years, and -0.13 m/s (p<0.001) per decade at 70 years, after adjusting for sex and height. No sex difference of gait speed decline was observed after adjustment for height. Time to finish Trails B, an indicator of executive function, increased 11.3 seconds per decade at 60 years, 17.7 seconds (p<0.001) per decade at 65 years, and 24.1 seconds (p<0.001) per decade at 70 years, after adjusting for sex, education, and race. No sex difference of longitudinal decline in executive function was observed. Linking these findings to physiological measures may unveil an important mechanism of aging.

